# Artificial Intelligence based wrapper for high dimensional feature selection

**DOI:** 10.1186/s12859-023-05502-x

**Published:** 2023-10-18

**Authors:** Rahi Jain, Wei Xu

**Affiliations:** 1 Biostatistics Department, Princess Margaret Cancer Research Centre, Toronto, ON Canada; 2 Dalla Lana School of Public Health, University of Toronto, Toronto, ON Canada

**Keywords:** High dimensional data, Wrapper feature selection, Artificial intelligence, AIWrap, Machine learning, Interaction terms

## Abstract

**Background:**

Feature selection is important in high dimensional data analysis. The wrapper approach is one of the ways to perform feature selection, but it is computationally intensive as it builds and evaluates models of multiple subsets of features. The existing wrapper algorithm primarily focuses on shortening the path to find an optimal feature set. However, it underutilizes the capability of feature subset models, which impacts feature selection and its predictive performance.

**Method and Results:**

This study proposes a novel Artificial Intelligence based Wrapper (AIWrap) algorithm that integrates Artificial Intelligence (AI) with the existing wrapper algorithm. The algorithm develops a Performance Prediction Model using AI which predicts the model performance of any feature set and allows the wrapper algorithm to evaluate the feature subset performance in a model without building the model. The algorithm can make the wrapper algorithm more relevant for high-dimensional data. We evaluate the performance of this algorithm using simulated studies and real research studies. AIWrap shows better or at par feature selection and model prediction performance than standard penalized feature selection algorithms and wrapper algorithms.

**Conclusion:**

AIWrap approach provides an alternative algorithm to the existing algorithms for feature selection. The current study focuses on AIWrap application in continuous cross-sectional data. However, it could be applied to other datasets like longitudinal, categorical and time-to-event biological data.

**Supplementary Information:**

The online version contains supplementary material available at 10.1186/s12859-023-05502-x.

## Background

Large feature space ($p$) is an important aspect of high dimensional data owing to model overfitting risk, poor generalizability [1] and computational complexity [2, 3]. Feature selection is a solution which reduces the input feature space to a smaller feature space ($q$) in a given dataset of sample size ($n$), which provides a parsimonious best fit model for the outcome, $y$.

1 $$y = f\left( q \right) | q \in \left( p \right)$$

where, $f$ represents the model function such that the error function $\varphi$ is minimized, i.e., $\mathrm{min}\varphi (y,f\left(q\right))$. The approaches adopted for feature selection can be categorized into two groups. The first and simpler approach uses expert opinion for feature selection where features are selected using domain knowledge [4, 5] and allows feature selection before data evaluation. This approach has limited or no applicability if a feature has no or little availability of domain information, high dimensional feature space and/or presence of interactions among the features.

The second approach uses the data to perform the feature selection. The algorithms under this approach are broadly classified into filter, embedded and wrapper algorithms [6–8] and could be used in supervised, semi-supervised or unsupervised learning frameworks [8–10]. Filter algorithms rely on the internal data structure of the features for selecting features. Commonly, information gain based algorithms are used for univariate filtering of features [8, 11] and correlation based algorithms are used for multivariate filtering of features [12]. They are computationally efficient, but interactions between the features may hinder the feature selection performance. Embedded algorithms incorporate feature selection within the model building step by adding a penalization step in the model building process. They are efficient and can handle interactions between the features. Least Absolute Shrinkage and Selection Operator (LASSO) based algorithms [13, 14] are commonly used for linear combination models, while tree-based algorithms [15] are used in non-linear combination models. Wrapper algorithms use an iterative approach of evaluating a feature subset for model performance on a given dataset. The process is repeated until the best performance is obtained [16, 17]. It provides better performance than other algorithms, but it has a higher computational cost.

The key challenge in wrapper algorithms is that models are prepared for every feature subset obtained at each iteration. One strategy is to reduce the number of iterations needed to get the target feature set $q$ for addressing the computational cost issue by focusing on the sampling of feature subset. Feature subset sampling step is commonly performed using either random, sequential or evolutionary sampling. The random sampling approach arbitrarily generates the feature subset [18]. The sequential sampling approach adds or removes a feature sequentially from a feature set like forward sampling and backward sampling [16, 19]. The evolutionary sampling approach selects the feature subset based on the performance of features in the previous subset like genetic algorithm [20] and swarm optimization [21]. Another strategy is to use hybrid algorithms, model building at the iteration step is replaced with filter techniques to estimate the performance of many feature subsets at each iteration step [22–24]. However, filter algorithm challenges persist.

We propose a unique strategy which uses an Artificial Intelligence (AI) model instead of filter techniques. Currently, the existing wrapper algorithms partially or entirely discard the unselected models of feature subset in selecting the next population of feature subsets. Individually, each model may only be useful in providing performance information, but in combination, these models could help in identifying hidden relationships that could predict the performance of unknown feature subset models. This eliminates the need for building models for every single feature subset obtained in the sampling step.

In this study, we propose a novel Artificial Intelligence based Wrapper (AIWrap) algorithm. The algorithm predicts the performance of unknown feature subset using an AI model referred here as Performance Prediction Model (PPM). To determine the performance of unknown feature set, standard wrapper estimates the performance by building a model of unknown feature set on the given dataset to calculate the actual model performance. AIWrap predicts the performance by creating PPM that uses the performances of known feature subsets to compute the performance of unknown feature subset.

AIWrap contributes in many ways. Firstly, it is unique in its perspective as, unlike standard wrapper approach of building models for every feature subset provided by feature subset sampling step, it builds models for only a fraction of the feature subset. Secondly, it provides a unique application of AI models, that are used to replace the AI model-based performance estimation step with AI model-based performance prediction step. Thirdly, AIWrap is versatile, which allows its integration with existing statistical and machine learning techniques. Fourthly, the algorithm allows the explicit identification of interaction terms.

This paper provides the “Results” section to evaluate and compare the algorithm performance against the existing feature selection algorithms for simulations and real studies. We summarize and provide future directions for research in the “Discussion” and “Conclusion” section. We provide “Conceptual Framework” section to explain the basic framework of AIWrap. Finally, the “Methodology” section explains the AIWrap algorithm used in this paper.

## Results

The performance of AIW rap is evaluated and compared with standard algorithms like LASSO, Adaptive LASSO (ALASSO), Group LASSO (GLASSO), Elastic net (Enet), Adaptive Elastic net (AEnet) and Sparse Partial Least Squares (SPLS) for both the simulated datasets and real data studies.

### Simulation studies

We perform simulation studies to evaluate the proposed algorithm and compare its performance with other feature selection algorithm. The study uses multivariate normal distributions to generate high-dimensional datasets for marginal and interaction models. The regression model, $y= {\beta }_{0}+ \sum_{i=1}^{p}{\beta }_{i}{x}_{i}+\epsilon$ and $y= {\beta }_{0}+ \sum_{i=1}^{p}{\beta }_{i}{x}_{i}+ \frac{1}{2}\sum_{i\ne j, i=1,j=1}^{i=p, j=p}{\beta }_{ij}{x}_{ij}+\epsilon$ provides the outcome variable of the simulated data for marginal and interaction models, respectively. Error term, $\varepsilon \sim N(0, {\sigma }^{2})$ and features, ${x}_{i} \sim N(0, 1)$ follow normal distribution. {${x}_{ij}$} represents the pairwise interactions between features $\{({x}_{1},{x}_{2}), \dots ,({x}_{2}, {x}_{3}), \dots , ({x}_{p-1},{ x}_{p})\}$. In the current study, only two-way interactions are considered for demonstration purposes, but it could be easily extended to higher-order interactions. Correlation is added between the first 15 features out of $p$ marginal features using the covariance matrix as given below.

$$\left[\begin{array}{ccc}\begin{array}{cc}{x}_{1}{x}_{1}& .\\ .& .\\ {x}_{15}{x}_{1}& .\end{array}& \begin{array}{cc}{x}_{1}{x}_{15}& .\\ .& .\\ {x}_{15}{x}_{15}& .\end{array}& \begin{array}{cc}.& {x}_{1}{x}_{p}\\ .& .\\ .& {x}_{15}{x}_{p}\end{array}\\ \begin{array}{cc}.& .\\ {x}_{p}{x}_{1}& .\end{array}& \begin{array}{cc}.& .\\ {x}_{p}{x}_{15}& .\end{array}& \begin{array}{cc}.& .\\ .& {x}_{p}{x}_{p}\end{array}\end{array}\right]=\left[\begin{array}{ccc}\begin{array}{cc}1& .\\ .& .\\ 5& 5\end{array}& \begin{array}{cc}5& .\\ 5& .\\ 1& .\end{array}& \begin{array}{cc}.& 0\\ .& .\\ .& 0\end{array}\\ \begin{array}{cc}.& .\\ 0& .\end{array}& \begin{array}{cc}.& .\\ 0& .\end{array}& \begin{array}{cc}.& .\\ .& 1\end{array}\end{array}\right]$$

Multiple scenarios are created with the different number of noise features (Table 1). Non-zero $\beta$ value is assigned only to the true features. The AIWrap algorithm is implemented both with and without a performance-based filter step. The final predictive model from selected features is prepared using either ridge regression (AIWrap-LR) or non-penalized linear regression (AIWrap-LLr). When no performance-based filter step is performed, model obtained from embedded feature selection stage is used as the final predictive model and is referred to as AIWrap-L technique. All penalized regression models used in AIWrap performed tenfold cross validation-based optimization of hyper-parameters to reduce overfitting.

**Table 1:** Description of the simulation data

Models	Scenario	$\beta$(Non-Zero coefficients)	$p$	Sample size ($n$)		$\sigma$
				Train	Test	
Marginal	1_M	$\{ \beta_{i} | i = \left\{ {1, \ldots ,10} \right\}\} =$ $\left\{ {0.5, - 0.5,0.5, - 0.5, \ldots , 0.5} \right\}$	50	50	500	0.25
	2_M		50	100	500	0.25
	3_M		100	75	500	0.25
	4_M		100	100	500	0.25
Interactions	1_I	$\{ \beta_{i} , \beta_{ij} | i = \left\{ {1, \ldots ,10} \right\}, j = i + 1, j < 11\} =$ $\left\{ {0.5, - 0.5,0.5, - 0.5, \ldots , 0.5} \right\}$	15	100	500	0.25
	2_I		25	100	500	0.25
	3_I		50	100	500	0.25

### Computation time estimation

AIWrap algorithm time complexity can change based on the techniques used to perform feature subset sampling, PPM and feature subset model building. In the current paper, LASSO, random forest and genetic algorithm are used and the time complexity is $$\mathrm{O}(\mathrm{g}*\mathrm{pop}*({p}^{3} + {\mathrm{k}}_{w}\mathrm{log}({\mathrm{k}}_{w})))$$ where $$g$$ is the number of generations in genetic algorithm, $$pop$$ is the population size in each generation, $$p$$ is the number of features and $${k}_{w}$$ is number of LASSO models used to train PPM (Additional File 1).Still, time complexity is also estimated using computation time of the AIWrap algorithm under different scenarios. The algorithm is run on a system with processor Intel® Core (TM) i7-8750H CPU@2.20 GHz with 16 GB RAM on a Windows 10 64-bit operating system. AIWrap algorithm is compared with a Standard Wrapper (StW) algorithm and hybrid algorithm. In StW, sampling method and feature subset model building method is same as AIWrap but does not have PPM and performance-based feature selection step. Further, an embedded feature selection step is added in StW. AIWrap-L version of AIWrap algorithm is used for comparison, thus any performance difference would be due to PPM. Genetic algorithm is used to generate samples in feature subset sampling step with maximum number of iterations fixed to 100.

Two methods are used in hybrid algorithms. Interaction Information-Guided Incremental Selection (IGIS) method is used as a sample hybrid algorithm for comparison [22]. This method uses sequential forward selection as wrapper feature sampling technique. Since, it is designed for classification problem, the filters used are mutual information and joint mutual information. Accordingly, the continuous outcome is converted into 10 equally spaced bins. A Modification of IGIS (mIGIS) is also used where filters used are correlation and ridge regression to allow the use of continuous outcome. Since, IGIS is not designed to provide explicit interaction terms, hybrid algorithms are not tested for scenarios containing interaction terms.

Multiple scenarios are created for the comparative analysis of algorithms (Table 1). The training datasets vary from 50 to 100 samples, while the test datasets contain 500 samples. In each scenario, training samples and test samples are independent samples that came from same distribution. Along with computation time, we evaluate algorithms on their ability to select the target features and predictive performance of selected features. F1 score is used to determine the accuracy of selecting target features. Root Mean Square Error (RMSE) from the test data is used to determine the predictive performance of the model obtained from the embedded feature selection step. RMSE on test data would also help in comparing the overfitting problem of different algorithms. All the analysis is conducted using R 4.0.3 [25].

In both the marginal and interaction models (Table 2), AIWrap has better or at par ability to discriminate between the target and noise features, especially for interaction models as compared to other algorithms. The similar performance of StW compared to hybrid methods could be the use of different search strategy. Similarly, predictive performance of the features shortlisted from AIWrap is better or at par with other algorithms, especially for high dimensional data and interaction models. AIWrap performance suggests that this methodology framework can be used as an alternative to the standard wrapper and hybrid framework.

**Table 2:** Algorithms comparison of computation time, target feature selection and predictive performance

*Model*	*p*	*n*	Performance											
			Target feature selection (F1 Score)				Predictive performance (RMSE)				Computation time (minutes)			
			*StW*	*IGIS*	*mIGIS*	*AIWrap*	*StW*	*IGIS*	*mIGIS*	*AIWrap*	*StW*	*IGIS*	*mIGIS*	*AIWrap*
1_M	50	50	**0.48**	0.00	0.18	0.47	0.55	1.14	1.14	**0.43**	**7.57**	< 0.02	0.07	24.85
2_M	50	100	0.71	0.00	**0.95**	0.63	0.29	1.11	0.30	**0.29**	**10.68**	< 0.02	0.60	23.93
3_M	100	75	0.29	0.00	**0.75**	**0.33**	0.64	1.19	0.81	**0.48**	**11.30**	< 0.02	0.62	18.22
4_M	100	100	0.42	0.18	0.18	**0.43**	0.36	1.18	1.18	**0.36**	**31.52**	< 0.02	0.12	33.07
1_I	15	50	0.41			**0.73**	1.20			**0.38**	**0.97**			2.18
2_I	25	50	0.26			**0.39**	1.32			**0.49**	**2.72**			7.23

Values in Bold means best results

The number of iterations in the genetic algorithm is predefined for both StW and proposed algorithm which indicates that proposed algorithm used lesser number of models to achieve the better outcome. However, AIWrap consumed more time as compared to standard wrapper approach which is counter intuitive. The current approach uses random forest to update PPM model and uses LASSO to build the base model for the unknown feature subset in both StW and proposed algorithm. Random forest used for PPM took more time in each run compared to the Lasso model used to build the model because during each upgrade, sample size used for training PPM model increases. LASSO needs to build the model on a sample size of 50 or 100 but random forest needs to build a PPM model using at least 225 samples (Model 1_I) with sample size increasing during the execution of genetic algorithm.

### AIWrap comparison with standard algorithms

AIWrap performance is compared with existing standard penalized regression algorithms namely LASSO, ALASSO, GLASSO, Enet, AEnet and SPLS in ten different trials. GLASSO is used only for interaction models. All the analysis is conducted using R 4.0.3 [25]. The standard algorithms are run using the inbuilt packages in statistical language R. *glmnet* package [26] is used for most algorithms except GLASSO and SPLS for which *glinternet* [27] and *spls* [28] packages are used. Adaptive weights are obtained from ridge regression [29] for adaptive models. In the case of interaction models, all possible two-way interaction terms are created and entered the model.

Algorithms are evaluated on target feature selection and prediction performance. We evaluate their ability to discriminate between true and noise features by measuring the selection of true features and rejection of noise features. We use RMSE from the test data as the predictive performance metric.

**Fig. 1:**
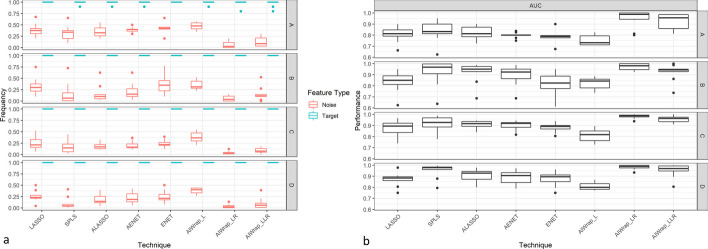
Comparison of different methods’ feature selection performance in marginal models **a** Frequency of selection of target and noise features. **b** AUC for predicting the target and noise features

Table 3 shows the feature selection performance of different algorithms for marginal models. All have selected the targeted ten features which means that they can identify the target features in the marginal dataset. However, in most cases, the number of selected features is much higher, indicating that methods also select noise features. AIWrap, compared to other algorithms, selected a similar or lesser number of noise features which suggests that it has better discrimination ability between noise and target features than standard methods (Fig. 1). It is shown that frequency of selecting a noise feature is consistently lesser than the target features in all methods, but the maximum separation is found only for AIWrap method. In addition, the area under curve (AUC) of the features was higher for AIWrap method as compared to standard methods. Thus, in the case of marginal datasets, while all methods can identify the target features, AIWrap outperforms all other methods with a lesser selection of noise features.

**Table 3:** Feature selection performance of different approaches in simulated scenarios for marginal models

Methods	Performance (Number of features selected)			
	Marginal Model Scenarios			
	*1_M*	*2_M*	*3_M*	*4_M*
	*p* = *50*	*p* = *50*	*p* = *100*	*p* = *100*
	*Target Features: 10*	*Target Features:* *10*	*Target Features:* *10*	*Target Features:* *10*
	*Mean (Range)*			
*ALASSO*	24 (18–32)	16 (11–35)	27 (20–39)	28 (14–46)
*LASSO*	25 (18–37)	23 (14–40)	32 (16–57)	33 (14–55)
*SPLS*	23 (14–35)	16 (10–39)	25 (12–50)	19 (11–47)
*Enet*	27 (18–36)	25 (14–41)	32 (21–45)	32 (17–55)
*AEnet*	26 (21–30)	18 (11–35)	28 (20–43)	30 (15–48)
*AIWRAP-L*	29 (24–33)	24 (19–31)	44 (29–59)	44 (34–51)
*AIWRAP-LLr*	15 (11–22)	16 (10–31)	18 (10–26)	19 (10–45)
*AIWRAP-LR*	**12** **(10–16)**	**12** **(10–16)**	**14** **(10–21)**	**13** **(10–22)**

Values in Bold means best results

**Fig. 2:**
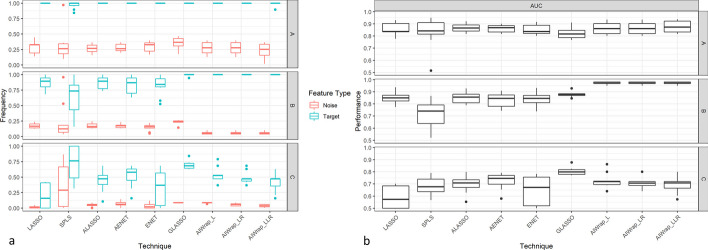
Feature selection performance comparison of different methods in interaction models **a** Frequency of selection of target and noise features. **b** AUC for predicting the target and noise features

The results from the interaction models reiterate the results of the marginal scenario that the feature selection performance of AIWrap is better or at par with the standard algorithms. Similar to marginal models, Table 4 shows that the number of features selected by all algorithms in interaction models is more than the number of target features in most cases. They all selected noise features, but the number of noise features selected differs with algorithm. Figure 2 suggests that AIWrap may be selecting a lesser number of noise features compared to other methods. In low dimensional space, all algorithms can discriminate between the target and noise features by selecting the target features at a higher frequency as compared to noise features. However, in very high dimensions, only AIWrap and GLASSO can perform. AUC performance of different methods also shows better or at par performance of AIWrap as it can predict the target and noise features with greater or similar accuracy than other methods.

**Table 4:** Feature selection performance of different approaches in simulated scenarios for interaction models

Methods	Performance (Number of Features Selected)					
	Interaction Model Scenarios					
	*1_M*		*2_M*		*3_M*	
	*p* = *15*	χ = 105	*p* = *25*	χ = 300	*p* = *50*	χ = 1225
	*Target Features*					
	*10*	*9*	*10*	*9*	*10*	*9*
	*Mean (Range)*					
*ALASSO*	15 (15–15)	31 (20–41)	24 (22–25)	46 (32–67)	32 (2–45)	36 (1–67)
*GLASSO*	15 (14–15)	40 (22–51)	25 (24–25)	66 (39–74)	47 (45–49)	76 (72–81)
*LASSO*	15 (15–15)	33 (18–49)	24 (22–25)	45 (30–65)	**16** **(1–45)**	**16** **(0–71)**
*SPLS*	14 (12–15)	36 (16–102)	19 (9–25)	65 (6–287)	38 (6–50)	417 (1–1057)
*Enet*	15 (15–15)	34 (21–44)	22 (14–25)	39 (11–60)	29 (2–50)	36 (1–116)
*AEnet*	15 (15–15)	32 (24–41)	24 (22–25)	44 (31–64)	37 (2–49)	53 (1–104)
*AIWRAP-L*	**12** **(12–14)**	34 (20–47)	18 (14–21)	50 (26–60)	29 (27–32)	85 (71–99)
*AIWRAP-LLr*	**12** **(12–14)**	**30** **(8–44)**	**16** **(10–20)**	**36** **(5–47)**	24 (8–30)	30 (2–52)
*AIWRAP-LR*	**12** **(12–14)**	34 (20–47)	18 (14–21)	50 (26–60)	28 (24–30)	46 (26–88)

Values in Bold means best results

AIWrap uses existing classic statistical techniques. The statistical techniques could influence the wrapper method performance [30]. However, a performance comparison between LASSO used in AIWrap and as a standalone feature selection algorithm clearly showed that AIWrap could improve the LASSO performance. The AIWrap performance suggests that the proposed algorithm could enhance the feature selection performance of the existing statistical methods by reducing the feature space and increasing the target feature percentage.

Table 5 shows the prediction performance of algorithms. RMSE performance suggests that AIWrap performs consistently better or at par with the existing algorithms. In low dimensionality data (2_M, 4_M and 1_I), it is expected that all algorithms should give similar performance as standard algorithms are primarily developed for handling low dimensionality data, and results support it. AIWrap can provide better performance even in high dimensional settings (1_M and 3_M) and in the presence of interaction terms (2_I). However, at very high dimensional data (3_I), all methods perform poorly. These findings suggest that the AIWrap may provide better or at par prediction performance than existing algorithms. Overall, the proposed algorithm could expand the capability of existing methods like non-penalized regression to operate in high-dimensional settings. However, computational intensiveness will be a significant limitation for the proposed algorithm compared to standard methods. In summary, performance of all algorithms deteriorates with an increase in data dimensionality, but performance of most standard methods decreases more drastically than AIWrap.

**Table 5:** Outcome prediction performance of different approaches in simulated scenarios for the test dataset

Methods	Performance (RMSE)						
	Marginal Model Scenarios				Interaction Model Scenarios		
	*1_M*	*2_M*	*3_M*	*4_M*	*1_I*	*2_I*	*3_I*
	*Mean (95% Confidence Interval)*						
*ALASSO*	0.44 (0.35–0.54)	0.28 (0.23–0.33)	0.39 (0.32–0.46)	0.30 (0.26–0.35)	0.44 (0.36–0.52)	0.94 (0.74–1.13)	1.36 (1.31–1.41)
*GLASSO*					0.36 (0.3–0.43)	0.65 (0.51–0.80)	**1.20** **(1.15–1.26)**
*LASSO*	0.45 (0.36–0.54)	0.29 (0.24–0.34)	0.40 (0.33–0.47)	0.31 (0.26–0.36)	0.40 (0.33–0.47)	0.94 (0.76–1.13)	1.36 (1.32–1.40)
*SPLS*	0.45 (0.35–0.55)	0.26 (0.21–0.31)	0.43 (0.28–0.58)	0.27 (0.23–0.31)	0.52 (0.38–0.66)	1.33 (1.21–1.45)	1.47 (1.38–1.56)
*Enet*	0.45 (0.36–0.53)	0.29 (0.24–0.35)	0.42 (0.34–0.5)	0.32 (0.27–0.36)	0.41 (0.34–0.49)	1.02 (0.82–1.22)	1.34 (1.29–1.38)
*AEnet*	0.46 (0.35–0.57)	0.28 (0.23–0.33)	0.41 (0.33–0.48)	0.31 (0.26–0.35)	0.46 (0.38–0.54)	0.97 (0.79–1.15)	1.34 (1.30–1.39)
*AIWRAP-L*	0.51 (0.38–0.65)	0.28 (0.23–0.32)	0.43 (0.34–0.52)	0.31 (0.26–0.36)	**0.36** **(0.29–0.43)**	**0.50** **(0.40–0.61)**	1.43 (1.30–1.57)
*AIWRAP-LLr*	**0.41** **(0.26–0.56)**	**0.26** **(0.21–0.31)**	**0.33** **(0.27–0.39)**	**0.27** **(0.22–0.32)**	0.39 (0.31–0.48)	0.58 (0.39–0.77)	1.44 (1.33–1.55)
*AIWRAP-LR*	0.46 (0.33–0.58)	0.30 (0.26–0.33)	0.34 (0.30–0.38)	0.29 (0.26–0.33)	0.56 (0.48–0.65)	0.79 (0.68–0.91)	1.35 (1.28–1.41)

Values in Bold means best results

### Real studies: population health data

Four real studies are analyzed to evaluate the performance of AIWrap and existing algorithms. Community Health Status Indicators (CHSI) study focuses on non-communicable diseases from US county with data (*n* = 3141) containing 578 features [31] (Study I). National Social Life, Health and Aging Project (NSHAP) datasets focusing on the health and well-being of aged Americans contains multiple datasets. We chose two datasets (Study II and Study III) containing data for 4377 residents on 1470 features [32] and 3005 residents on 820 features [33]. Study IV is the Study of Women’s Health Across the Nation (SWAN), 2006–2008 dataset focusing on 887 *physical, biological, psychological and social* features in middle-aged women in the USA (*n* = 2245) [34].

The raw data of the real studies are processed for ease of analysis to obtain final datasets (Table 6). Features and samples are filtered to remove highly correlated features, non-continuous features, and missing values. Then, each dataset is randomly split into training and testing datasets. As the sample size is large, only 20% of data is used for training while remaining 80% of data is used for testing to create a high dimensional data setting. We compare the performance of different algorithms for marginal models and interaction models using mean RMSE of the test data in ten trials.

**Table 6:** Summary of the real datasets

Real Studies	Marginal Features (p)	Outcome feature	Sample size (n)		
			Total	Train	Test
Study I	44	Percentage of unhealthy days	1471	294	1177
Study II	19	Height	1287	257	1030
Study III	33	Height	943	189	754
Study IV	26	Body Mass Index	1406	281	1125

Table 7 summarizes the feature selection results. It is shown that standard algorithms are selecting a lesser number of features as compared to AIWrap. However, the results from the previous simulated data studies suggest that standard methods may struggle to discriminate between target and noise features (Figs. 1 and 2). Further, the predictive performance results of AIWrap is better than the standard algorithms for both marginal as well as interaction models (Table 8). The better performance of the proposed algorithm suggests that it may be more reliable than standard algorithms in identifying the target features.

**Table 7:** Number of features selected by different wrapper methods on the real studies

Real Studies	Performance (Number of Features Selected)	Existing Models					AIWRAP		
		*ALASSO*	*GLASSO*	*LASSO*	*SPLS*	Enet	AEnet	*AIWRAP-L*	*AIWRAP -LLr*
		*Mean (Range)*							
		*Marginal Models*							
I	Marginal (p = 44)	**7** **(4–14)**		**7** **(3–16**)	23 (3–44)	13 (4–22)	11 (4–21)	13 (7–21)	10 (5–16)
II	Marginal (p = 19)	**5** **(1–10)**		7 (1–12)	9 (1–15)	8 (1–15)	7 (1–12)	9 (4–13)	6 (3–9)
III	Marginal (p = 33)	**8** **(4–11)**		12 (6–16)	11 (4–33)	13 (5–18)	10 (4–18)	13 (10–18)	9 (4–13)
IV	Marginal (p = 26)	6 (5–7)		7 (5–9)	7 (5–14)	8 (5–11)	7 (5–12)	7 (5–9)	**5** **(3–9)**
		***Interaction Models***							
I	Marginal (p = 44)	13 (7–24)	42 (41–43)	**12** **(7–23)**	**12** **(3–44)**	22 (10–36)	21 (7–32)	21 (15–26)	20 (14–26)
	Interaction (χ = 946)	**4** **(1–11)**	170 (156–183)	**4** **(0–11)**	63 (0–591)	13 (1–46)	11 (0–23)	23 (8–47)	17 (5–35)
II	Marginal (p = 19)	10 (2–18)	19 (19–19)	**9** **(1–16)**	11 (1–19)	**9** **(1–15)**	10 (1–16)	12 (9–15)	10 (1–14)
	Interaction (χ = 171)	6 (0–19)	94 (87–108)	**4** **(0–8)**	24 (0–117)	6 (0–21)	6 (0–14)	15 (5–37)	8 (0–13)
III	Marginal (p = 33)	15 (6–26)	33 (32–33)	15 (3–23)	**4** **(1–10)**	14 (4–23)	16 (10–23)	16 (10–21)	15 (2–21)
	Interaction (χ = 528)	6 (1–25)	125 (113–137)	5 (0–16)	**1** **(0–4)**	4 (0–16)	5 (1–15)	22 (1–49)	19 (1–49)
IV	Marginal (p = 26)	5 (3–6)	7 (5–9)	6 (3–9)	9 (6–12)	7 (4–10)	**5** **(3–6)**	10 (6–13)	10 (6–13)
	Interaction (χ = 299)	3 (1–4)	7 (5–10)	4 (2–6)	12 (7–16)	5 (2–7)	**3** **(1–5)**	13 (7–26)	13 (7–26)

Values in Bold means best results

**Table 8:** RMSE performance of different wrapper methods on the real studies for test data

Methods	Performance (RMSE)			
	Marginal Model Scenarios			
	*I*	*II*	*III*	*IV*
	*Mean (95% Confidence Interval)*			
*ALASSO*	0.95 (0.95–0.96)	3.76 (3.67–3.84)	3.08 (3.01–3.14)	0.86 (0.81–0.90)
*LASSO*	0.96 (0.95–0.97)	3.75 (3.65–3.85)	3.10 (3.03–3.16)	0.84 (0.8–0.87)
*SPLS*	0.97 (0.95–0.99)	3.61 (3.54–3.69)	3.35 (3.03–3.66)	0.77 (0.76–0.79)
*Enet*	0.95 (0.94–0.96)	3.79 (3.7–3.87)	3.15 (3.08–3.23)	0.85 (0.81–0.90)
*AEnet*	0.96 (0.94–0.97)	3.76 (3.67–3.85)	3.11 (3.07–3.15)	0.84 (0.8–0.87)
*AIWRAP-L*	**0.94** **(0.93–0.94)**	3.65 (3.59–3.71)	3.02 (2.98–3.06)	0.83 (0.8–0.86)
*AIWRAP-LLr*	0.96 (0.94–0.97)	**3.59** **(3.55–3.64)**	**2.97** **(2.91–3.03)**	**0.75** **(0.73–0.78)**
*AIWRAP-LR*	0.95 (0.94–0.96)	3.80 (3.72–3.87)	3.19 (3.11–3.28)	1.20 (1.17–1.24)
Methods	**Interaction Model Scenarios**			
	***I***	***II***	***III***	***IV***
	***Mean (95% Confidence Interval)***			
*ALASSO*	0.94 (0.93–0.95)	3.69 (3.61–3.76)	3.12 (3.02–3.23)	0.52 (0.49–0.55)
*GLASSO*	1.44 (1.2–1.68)	4.46 (4.35–4.57)	8.24 (5.37–11.11)	0.31 (0.28–0.34)
*LASSO*	0.95 (0.94–0.96)	3.74 (3.67–3.81)	3.15 (3.02–3.27)	0.43 (0.39–0.47)
*SPLS*	1.03 (0.91–1.15)	3.81 (3.76–3.86)	4.34 (3.26–5.42)	**0.24** **(0.22–0.26)**
*Enet*	0.94 (0.93–0.95)	3.78 (3.72–3.84)	3.24 (3.13–3.34)	0.44 (0.4–0.48)
*AEnet*	0.93 (0.92–0.94)	3.73 (3.65–3.81)	3.14 (3.06–3.21)	0.53 (0.5–0.56)
*AIWRAP-L*	0.94 (0.92–0.95)	**3.58** **(3.53–3.63)**	**3.07** **(2.98–3.17)**	0.29 (0.26–0.33)
*AIWRAP-LLr*	1.04 (0.99–1.1)	3.76 (3.58–3.93)	3.65 (3.26–4.04)	0.26 (0.21–0.31)
*AIWRAP-LR*	**0.93** **(0.92–0.94)**	3.70 (3.64–3.76)	3.22 (3.18–3.26)	1.11 (0.99–1.24)

Values in Bold means best results

The results show that in Study III, marginal models performed better than their interaction models for all algorithms. Better performance of the marginal model compared to the interaction model suggests that AIWrap cannot completely reject noise features and is sensitive to an increase in feature space. However, AIWrap is still more robust than standard algorithms and can perform in different dimensions and datasets.

### Real studies: genomic data

AIWrap-L algorithm is compared with StW in the genomic datasets to determine the biological relevance of the solutions obtained from AIWrap. In many cancer studies, it is found that smoking can be detrimental to the cancer patient health [35, 36]. Further, an association between gene expression levels and cancer patient smoking habit has been reported [37]. Thus, it would be relevant to identify the genes in cancer patients which are associated with smoking-related traits. In this study, The Cancer Genomic Atlas (TCGA) program is used to get the data from nine cancer projects (Table 9) which maintained records related to amount smoked and gene expression profile of patients [38]. The sample size $n$ for these projects range from 89 to 592 samples with feature space $p$ of 56,602 genes. The gene expression profile is used as the input feature space and number of cigarettes smoked per day (CPD) is used as the outcome.

**Table 9:** Summary of the genomic datasets

Datasets	Number of cigarettes smoked per day (µ(σ))	Sample size (n)	Feature space (p)
TCGA-BLCA	1.16 (2.34)	433	56,602
TCGA-CESC	0.30 (0.62)	307	56,602
TCGA-ESCA	0.95 (1.21)	172	56,602
TCGA-HNSC	1.41 (1.89)	544	56,602
TCGA-KICH	0.21 (0.67)	89	56,602
TCGA-KIRP	0.42 (1.04)	320	56,602
TCGA-LUAD	1.53 (1.59)	592	56,602
TCGA-LUSC	2.44 (1.88)	551	56,602
TCGA-PAAD	0.46 (0.88)	181	56,602

Preliminary processing of all datasets is performed to reduce the input feature space and remove samples with missing values. The input feature space is reduced from 56,602 to 50 features through multi-stage processing (Table 9). Step one involved removing the features which are not differentially expressed in cancer patients as compared to normal patients using *TCGAbiolinks* package [39]. Step two involved supervised dimensionality reduction of the differentially expressed genes using partial least squares technique and select top 100 features with highest absolute weights in first latent feature. Step three involved removing correlations among the features. Thus, among any pair of features with more than 0.8 absolute correlation, one feature is randomly selected. Step four involves selecting the top 50 features among the non-correlated features based on their absolute weight in the first latent feature obtained in step two. No interaction effects are considered for this analysis.

The performance of AIWrap and StW in all datasets is compared on three metrics namely predictive performance, computation time and number of genes selected. The results are based on tenfold cross-validation (Table 10). It observed that in all the datasets the predictive performance of AIWrap based features is better or at par with StW based features. Further, it is observed that a smaller set of features are selected by AIWrap as compared to StW which suggests AIWrap could provide a more parsimonious set of features as compared to StW without compromising on the predictive performance of the features. In terms of computation time, the results are similar to those observed in simulation studies with StW taking less time than AIWrap in most cases. The stability of AIWrap is similar to StW when compared using standard deviation of predictive performance (Additional file 2: Table S1).

**Table 10:** Wrapper methods comparison of predictive performance, number of genes selected and computation time

Dataset	Performance (µ [95% CI])					
	Predictive performance (RMSE)		Number of genes selected		Computation time (minutes)	
	*StW*	*AIWRAP-L*	*StW*	*AIWRAP-L*	*StW*	*AIWRAP-L*
TCGA-BLCA	0.79[0.31,1.27]	**0.78[0.30,1.26]**	4[0,9]	1[0,3]	**5.9[3.2,8.6]**	12.2[10.1,14.3]
TCGA-CESC	1.00[0.84,1.16]	**0.98[0.84,1.13]**	10[7, 13]	5[4, 6]	**11[7.7,14.2]**	14.6[9.9,19.3]
TCGA-ESCA	1.04[0.87,1.20]	**1.00[0.85,1.15]**	11[5, 17]	8[2, 14]	**7.2[4.9,9.5]**	27.9[3.6,52.2]
TCGA-HNSC	0.99[0.82,1.16]	**0.98[0.81,1.15]**	16[12, 20]	6[3, 9]	**11.4[8.7,14]**	20.3[9.3,31.2]
TCGA-KICH	1.03[0.61,1.46]	**0.82[0.39,1.25]**	11[9, 13]	6[4, 8]	50.2[24.7,75.7]	**10.6[7.5,13.7]**
TCGA-KIRP	0.95[0.66,1.24]	**0.95[0.65,1.24]**	19[18, 20]	15[11, 19]	**10.4[8.8,12]**	41.1[12.5,69.8]
TCGA-LUAD	1.02[0.93,1.11]	**1.02[0.94,1.09]**	25[22, 28]	21[16, 26]	**11.6[9.1,14.1]**	42.3[11.6,72.9]
TCGA-LUSC	0.99[0.91,1.08]	**0.99[0.91,1.08]**	2[1, 3]	1[0,2]	**5.7[4.4,7]**	12[8.8,15.2]
TCGA-PAAD	1.26[0.74,1.79]	**1.24[0.75,1.73]**	22[20, 24]	14[9, 19]	**10.8[7.6,14.1]**	29[0.6,57.4]

Values in Bold means best results

In order to assess the biological relevance of the genes selected by each method, selected genes of each dataset are pooled together to create final list of genes selected by each method. The results show that some genes are selected at a very high frequency in dataset during tenfold feature selection process. Genes need to fulfill one of the two criteria of either having highest selection frequency or selection frequency of more than 80%. Accordingly, across nine datasets, AIWrap provided 13 genes while StW provided 40 genes. 11 genes (VCX3A, WNT3A, CALHM5, ZMYND10, FOXE1, PLAT, BAAT, WFDC5, CGB5, FADD, APOE) are found to be common across the two methods. Based on the univariate of the genes selected from two algorithms, it is found that 9 out of 13 AIWRAP genes and 19 out of 40 StW genes are statistically significant. In multivariate analysis, it is found that 8 out of 13 AIWrap and 18 out of 40 StW genes are statistically significant (Additional file 2: Table S2). Among the 13 genes from AIWrap, seven genes (WNT3A [40], TMEM45A [41], BAAT [41], WFDC5 [42], HS3ST5 [43], CGB5 and APOE [44]) have been reported in literature to exert influence on tobacco or smoking-related traits. Further, AIWrap identified six new genes (VCX3A, CALHM5, ZMYND10, FOXE1, PLAT, FADD) which could be related to smoking in cancer patients, thus providing an opportunity for identifying previously unknown biological functions.

## Discussion

Building models for each sample feature set obtained during the feature sampling stage of wrapper methods consume computational resources and may not always provide the best results. AIWrap allows skipping the model building for many sample feature sets by training an AI model, i.e., PPM, which could predict the performance of sample feature sets. AIWrap feature selection performance and predictive performance are better or at par than both the standard wrapper method and penalized standard algorithms, namely LASSO, ALASSO, GLASSO, SPLS, Enet and AEnet.

The proposed algorithm has certain limitations. The current study primarily focuses on testing the concept; thus, the study performed testing on limited datatypes. Future research could focus on evaluating the robustness of the approach using different types of data such as temporal data and categorical data, and outcomes such as binary outcomes and time to event outcomes. Other than data types, the focus could also be directed towards the techniques used in the algorithm. Currently, the study uses a linear combination function for building actual models, but future studies could also explore the non-linear combination function for model building. Further, the current study reduced the need to build actual models in the wrapper approach but could not eliminate it. Therefore, future research could use other PPM building techniques like an artificial neural network and support vector machines to eliminate the need for actual models. Finally, time complexity is a challenge which could be explored in future research.

## Conclusion

In the paper, we propose AIWrap, an innovative algorithm to perform wrapper based feature selection. The algorithm is flexible enough to work with both marginal and interaction terms. The algorithm could be easily embedded with any of the wrapper techniques as it does not alter existing methods, which allows users to integrate the algorithm in their existing wrapper pipelines. This approach could enhance the performance of existing wrapper techniques available in the literature for high dimensional datasets by reducing the number of models needed to search space. AIWrap can identify both the marginal features and interaction terms without using interaction terms in PPM, which could be critical in reducing the feature space any pipeline has to process.

The benefits of AIWrap comes from using AI to learn the dataset performance behavior and build the PPM, which replaces the actual model building process. The studies involving marginal effects with and without interaction effects in simulated data showed that AIWrap could outperform existing algorithms in feature selection and prediction performance. Similar performance in real datasets also demonstrates the practical relevance of AIWrap.

### Conceptual framework

**Fig. 3:**
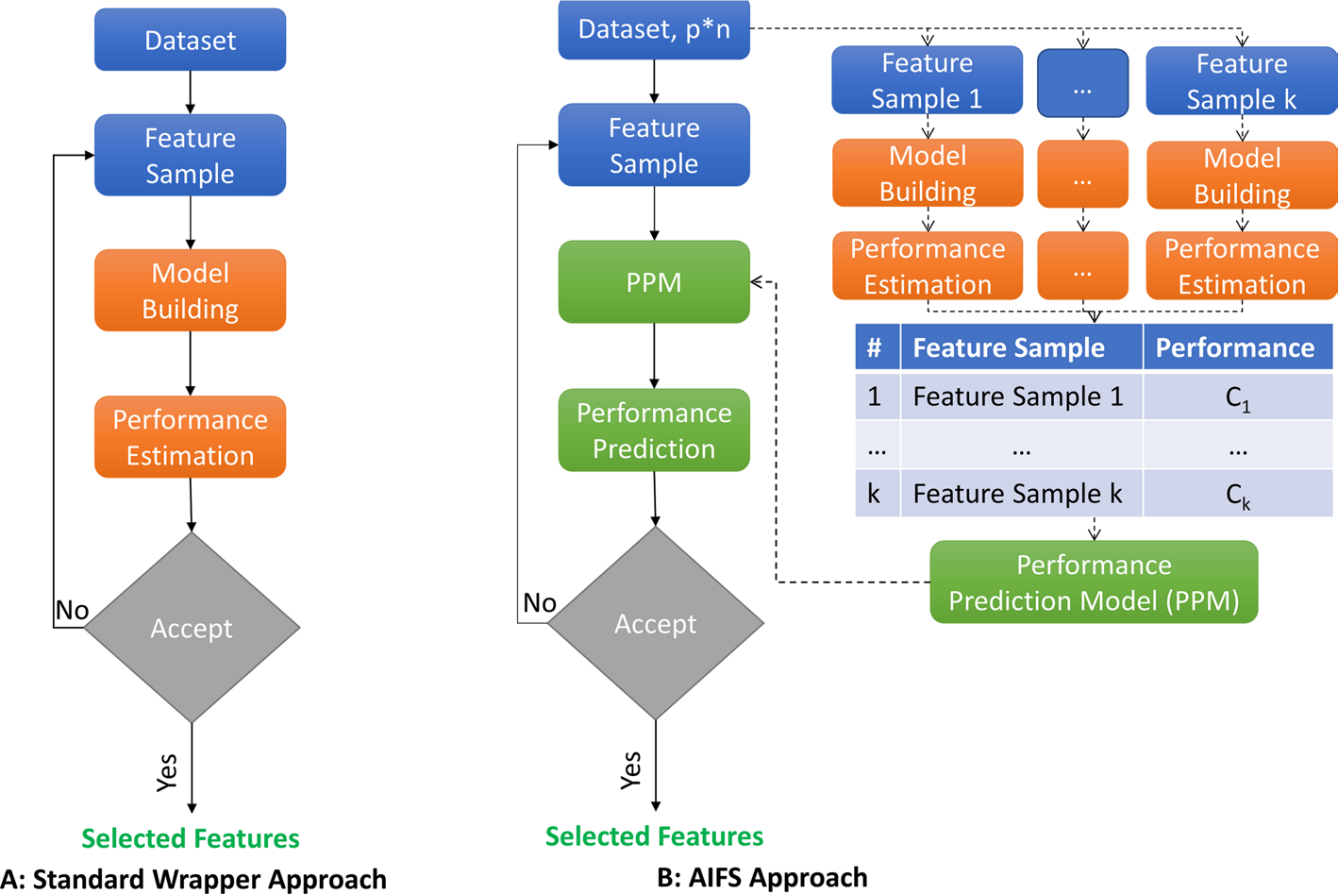
Flow chart of **A** Standard wrapper approach and **B** Proposed wrapper (AIWrap) conceptual approach

In a wrapper algorithm, given a dataset $D$ of sample size $n$ with $p$ feature space and outcome $y$, a subset feature set $q$ is created from $p$. In the standard wrapper algorithm (Fig. 3a), a model is built for the subset of $D$ containing $q$ features and performance is estimated by building model using the dataset. This performance is used to select the next subset of $p$. This dependence of a standard wrapper algorithms upon model building step for each subset of feature to estimate its performance is targeted in our AIWrap algorithm.

The conceptual framework used to design AIWrap algorithm (Fig. 3b) aims at reducing (or removing) the dependence of the wrapper algorithm on model building step for obtaining performance value of $q$. PPM will compute the unknown subset performance based on the known subsets performance. A user may not have a predefined list of known $k$ feature samples with their actual performance. AIWrap algorithm creates a random set ${q}_{AI}= \left\{ {q}_{{AI}_{j}}\right\}|{q}_{{AI}_{j}}\in \left\{\left\{1\right\},\dots ,\left\{1,\dots ,p\right\}\right\}, j\in \left\{1,\dots ,k\right\}$ of $k$ feature samples, where each feature sample is a subset of $p$. The algorithm builds a model for ${q}_{AI}$ samples to estimate their performance $C= \left\{{c}_{j}\right\}$. The algorithm creates PPM with ${q}_{AI}$ as the input and $c$ as the outcome using a machine learning model to enable performance prediction of any subset of $p$. Finally, the algorithm executes the standard wrapper approach, but uses PPM as a surrogate to the actual model building step that predicts rather than estimates the actual performance of $q$.

### Methodology

**Fig. 4:**
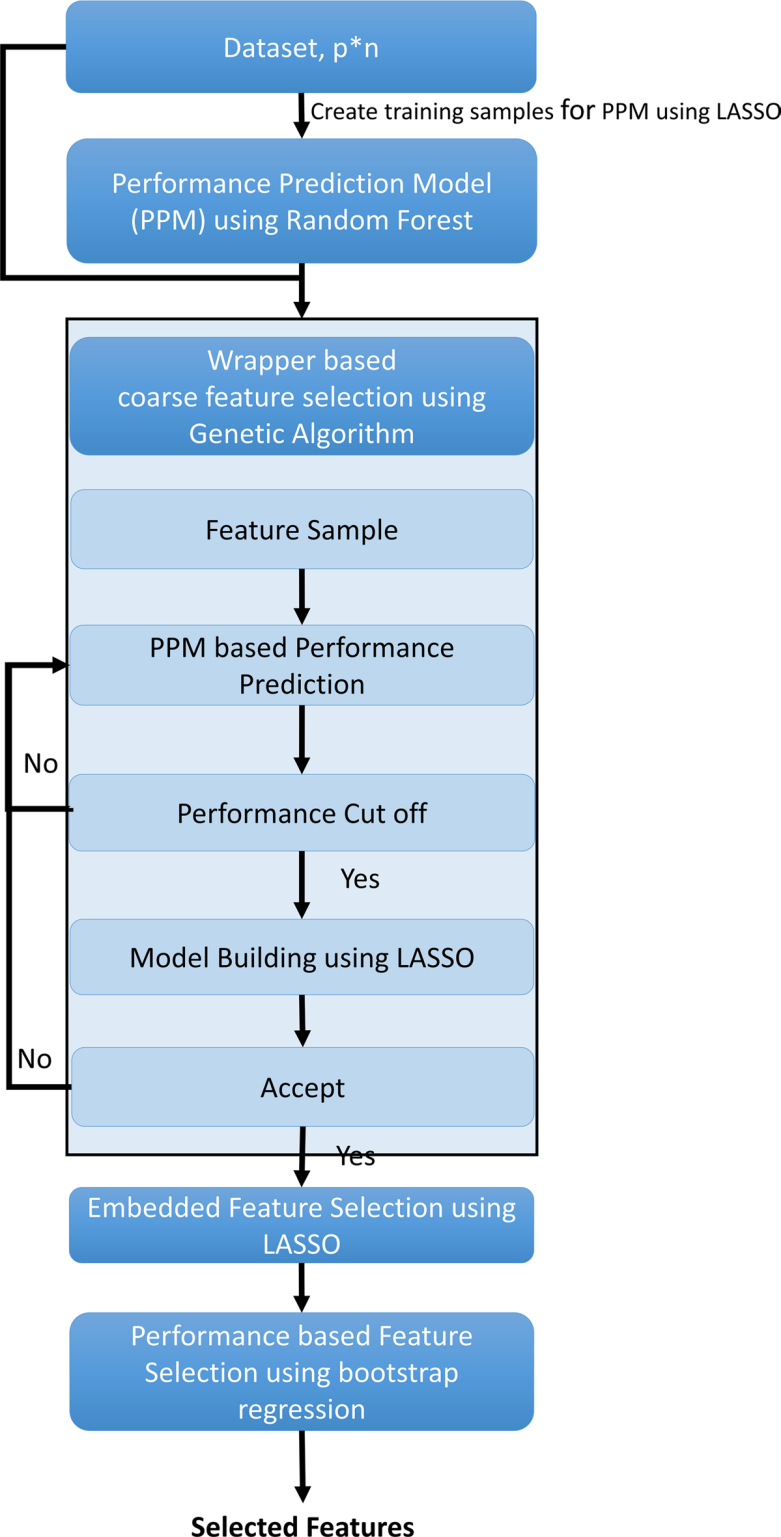
AIWrap algorithm graphical flow chart. Dark Background represents main steps and light background represents sub-steps

This section explains the design of AIWrap algorithm based on the conceptual framework. The algorithm is divided into four steps: PPM, wrapper based coarse feature selection, embedded-feature selection and performance-based feature selection (Fig. 4).

#### PPM

The algorithm generates $k$ random sample datasets containing ${q}_{{AI}_{j}}$ features, and sample size $n$ from$D$. A set of models $M= \left\{{m}_{j}\right\}$ are created from $k$ sample datasets for an outcome $, y$ using any modeling technique to obtain its performance,$c$. $k$ is a hyperparameter which user needs to optimize. In the current study,$k=15*p$, which is determined heuristically.

2 $$m_{j} : y_{j} = f\left( {q_{{AI_{j} }} } \right)| j \in \left\{ {1, \ldots ,k} \right\}$$

A performance set $C= \left\{{c}_{j}\right\}$ contains the performance of $M$ models. The algorithm creates a performance dataset ${D}_{perf}$, a matrix of features used in each model of $M$ (${q}_{f}$) and their performance, $C$.

3 $$D_{perf} = \left\| {\begin{array}{*{20}c} {q_{{f_{ij} }} } & {c_{j} } \\ \end{array} } \right\|q_{{f_{ij} }} = \left\{ {\begin{array}{*{20}c} {0, q_{{AI_{ij} }} \notin { }\left\{ {m_{j} } \right\} , i \in \left\{ {1,p} \right\},j \in \left\{ {1, \ldots ,k} \right\}} \\ {1, q_{{AI_{ij} }} \in { }\left\{ {m_{j} } \right\} , i \in \left\{ {1,p} \right\},j \in \left\{ {1, \ldots ,k} \right\}} \\ \end{array} } \right.$$

As shown in Eq. 3, feature matrix (${q}_{f}$) is a binary matrix that consists of $p$ columns and $k$ rows. The matrix takes the value of 0 for ${i}^{th}$ column and ${j}^{th}$ row, if ${i}^{th}$ feature is not used in ${m}_{j}$ model, else ${i}^{th}$ column and ${j}^{th}$ row takes the value of 1. PPM is constructed using any machine learning technique from ${D}_{perf}$ to predict performance, ${C}_{pred}$ of any unknown dataset.

4 $$PPM:C_{pred} = f\left( {q_{f} } \right)$$

In this study, we have used LASSO to prepare ${m}_{j}$ models and random forest to build the PPM with RMSE as the performance metric. During the preliminary analysis (Additional file 3), it is found that predicted performance and actual performance is strongly and positively correlated, but predicted performance may not match the actual performance, as a result subset corresponding to best predicted performance may not be the best subset.

### Wrapper based coarse feature selection

The standard wrapper algorithm as shown in Fig. 3a is an iterative process where a subset of feature is evaluated, and performance of the feature subset is used to select the next subset of features. In our work, we used genetic algorithm to search through the feature space iteratively as it is used in wide range of datasets [45, 46]. In the proposed algorithm, we use PPM for all iterations to predict the performance ${C}_{pred}$ of a feature set $q$. Since, we found that best ${C}_{pred}$ may correspond to one of the high performing feature sets but not the best feature set, we validate ${C}_{pred}$ values by building a model using $q$ features to estimate the performance ${C}_{true}$ (Fig. 4). The algorithm uses user-defined criteria ${val}_{crit}$ to select sample feature sets for validation of ${C}_{pred}$ values.

In this study, the top quartile of C is used as the ${val}_{crit}$ criterion, thus $q$ with ${C}_{pred}$ in top quartile of C are selected for model building. ${D}_{perf}$ is updated with feature set $q$ whose ${C}_{true}$ value is available and consequently, is used to update PPM. The iteration stops when we get ${q}_{wrap}$ features, which provide the best performance. RMSE is used as the performance metric.

### Embedded feature selection

The ${q}_{wrap}$ features obtained from the wrapper step are processed to obtain the final features because the prediction model does not explicitly provide the non-linear combinations of ${q}_{wrap}$ features. Thus, an embedded feature selection model is used on ${q}_{wrap}$ features for an outcome $, y$ which allows the additional features χ like interactions terms to be incorporated. LASSO framework is used as the embedded model in the proposed algorithm. LASSO hyper-parameters are optimized using tenfold cross validation of training data to reduce overfitting issue of the algorithm (Additional file 3).

### Performance-based feature selection

The features selected from the embedded model ${q}_{embed}$ undergo the last stage of processing to provide final features $q$. This step selects features based on their contribution to the model performance. $l$ models ${{m}_{{perf}_{l}}: y}_{j}=f\left({q}_{embed}-l\right)| l\in \left\{1,\dots ,{q}_{embed}\right\}$ are prepared with each model containing ${q}_{embed}-1$ features. $l$ feature importance is determined from the ${m}_{{perf}_{l}}$ performance.

To obtain $l$ feature robust importance, we create multiple models using bootstrapping of samples, and their performance ${\widehat{c}}_{j}$ is pooled to get overall model performance ${\widehat{c}}_{{pool}_{j}}$. In this study, we use ridge regression for model building as we are focusing on high dimensional data and non-penalized linear regression could only work for cases with ${q}_{embed}<n$. Goodness of fit (${R}^{2}$) of out of the bag (OOB) samples is used as the performance metric. Finally, the performance metric is pooled to provide a coefficient of variation of ${R}^{2}$ as the overall model performance for $l$ feature.

A performance threshold ${c}_{cutoff}$ needs to be defined to select the features. Rather than using an arbitrary threshold, our algorithm uses a dynamic cutoff. The algorithm tries different performance thresholds and selects the threshold which provides the best performance ${c}_{best}$ for the smallest feature space ${q}_{best}$. In the current study, we use genetic algorithm to search through the performance threshold space. Two different techniques, namely non-penalized regression and adaptive ridge regression are used for the model building. Pseudo Algorithm summarizes the complete AIWrap algorithm.
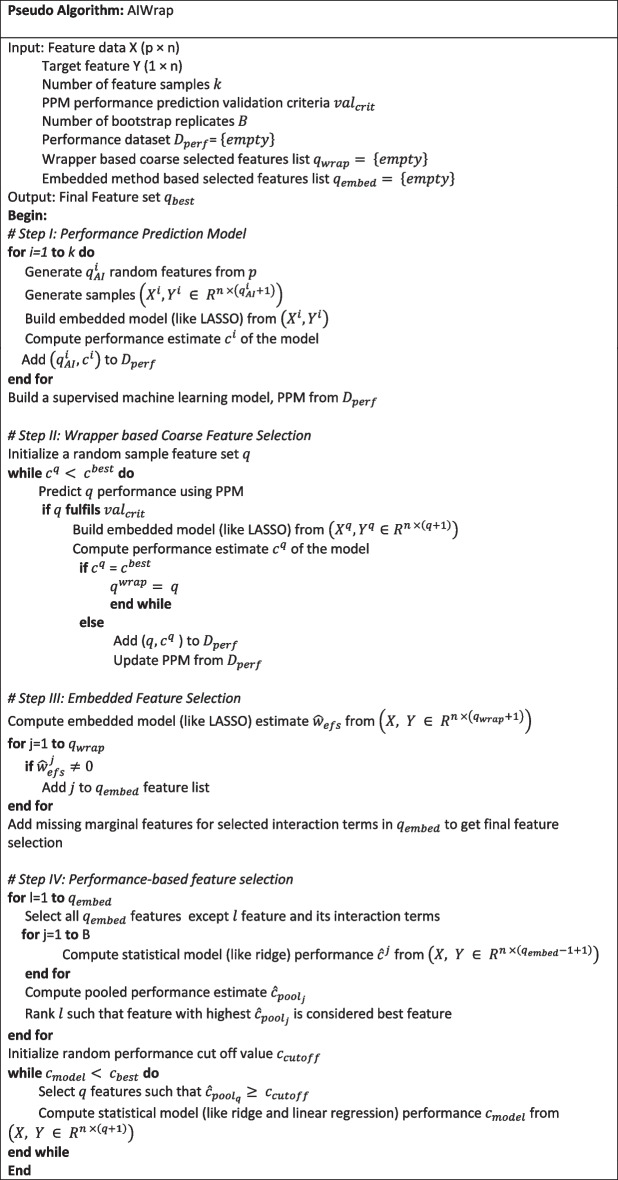


### Supplementary Information

**Additional file 1.** Time complexity estimation of AIWrap.

**Additional file 2.** Performance of StW and AIWrap in genomic dataset.

**Additional file 3.** Preliminary analysis of Random Forest.

## Data Availability

The code is in the GitHub link: https://github.com/rahijaingithub/AIWrap/tree/main. More details could be provided on request.

## Abbreviations

AEnet: Adaptive Elastic net
AI: Artificial Intelligence
AIWrap: Artificial Intelligence based wrapper
ALASSO: Adaptive LASSO
AUC: Area under curve
CHSI: Community Health Status Indicators
Enet: Elastic net
GLASSO: Group LASSO
NSHAP: National Social Life, Health and Aging Project
OOB: Out of the bag
PPM: Performance Prediction Model
RMSE: Root Mean Square Error
SPLS: Sparse Partial Least Squares
StW: Standard wrapper
SWAN: Study of Women’s Health Across the Nation
