# Supplementary material for: Artificial Intelligence based wrapper for high dimensional feature selection
Source: BMC Bioinformatics. 2023 Oct 18;24:392. doi: 10.1186/s12859-023-05502-x (PMC10585895; doi:10.1186/s12859-023-05502-x)
Supplement: Supplementary file 1 — Additional file 1. Time complexity estimation of AIWrap. [file 12859_2023_5502_MOESM1_ESM.docx]

Time Complexity Assessment

AIWrap has three main steps:

1. Sample Model Preparation: LASSO is used for building models. Lasso time complexity is $O(p^{3}+ p^{2}n)$where p is the number of features in the dataset including interaction terms and n is the sample size. This step is performed k times, which is a function of marginal features, m. Assuming m <<p and n<p, the time complexity of the step is $O(p^{3})$.
2. PPM: Random Forest is used to build the model. Random forest time complexity with no pruning is $O(k^{'}*k\log k*m)$ where k’ is number of trees. Assuming k >>k’ and k >>m, the time complexity is $O(k\log k)$.
3. Wrapper based feature selection: Genetic algorithm is used as the wrapper with fitness function incorporating PPM based prediction, PPM upgradation and LASSO based validation. Assuming a known and fixed mutation and cross over operators, the genetic algorithm time complexity is O(g*pop*O(fitness)) where g is the number of generations and pop is the population size in each generation. Assuming that PPM based prediction << PPM upgradation, then O(fitness) = O(Random forest + LASSO). In worst case scenario, the number samples used to retrain PPM model will increase after each LASSO model, thus $k_{w}=k+g*pop[1+g*pop]$. Thus, the time complexity is $O(g*pop*p^{3}+ k_{w}\log k_{w})$.

Assuming the wrapper to be the main step, thus the final time complexity of the model is $O(g*pop*p^{3}+ k_{w}\log k_{w})$.
