# Supplementary material for: Artificial Intelligence based wrapper for high dimensional feature selection
Source: BMC Bioinformatics. 2023 Oct 18;24:392. doi: 10.1186/s12859-023-05502-x (PMC10585895; doi:10.1186/s12859-023-05502-x)
Supplement: Supplementary file 3 — Additional file 3. Preliminary analysis of Random Forest. [file 12859_2023_5502_MOESM3_ESM.docx]

Plot of true performance vs predicted performance for the dataset with 50 features and 50 samples.


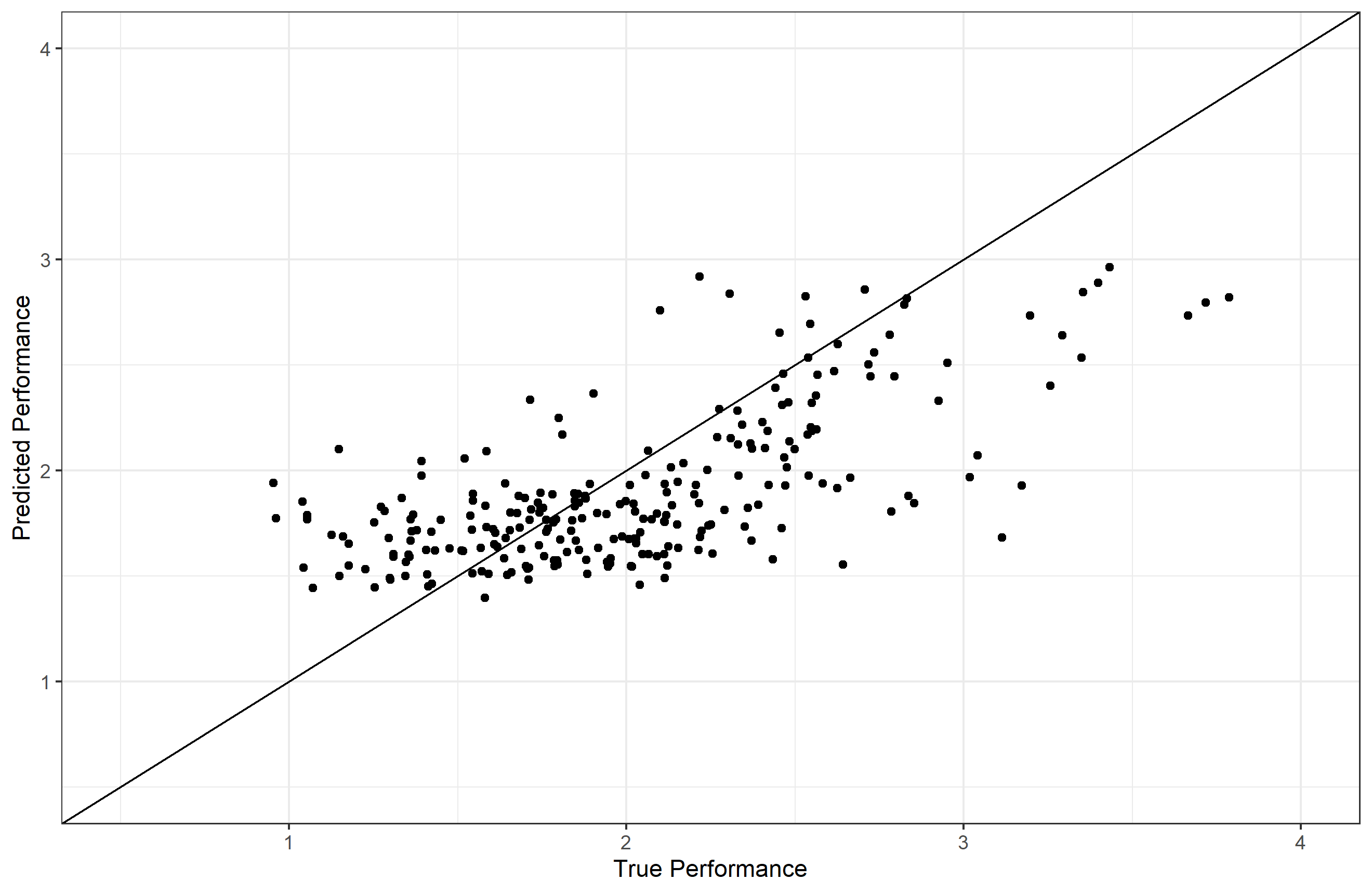


Appendix II:

Hyperparameters used in the AIFS algorithm are as follows:

| **#** | **Hyperparameter** | **Value** |
| --- | --- | --- |
| ***Performance Prediction Model (PPM)*** | | |
| 1 | k | 15n |
| ***Wrapper based coarse feature selection*** | | |
| 2 | Number of additional q added to dataset D to initiate PPM model retraining | 50 |
| ***Performance-based feature selection*** | | |
| 3 | Number of Bootstraps, B | 100 |
